# Heterogeneous course of diabetic polyneuropathy in type 1 diabetes over 5 years: is regression possible?

**DOI:** 10.1007/s12020-026-04613-8

**Published:** 2026-04-27

**Authors:** Pietro Pertile, Ilenia D’Ippolito, Sara Mascambroni, Cinzia D’Amato, Aikaterini Andreadi, Davide Lauro, Vincenza Spallone

**Affiliations:** Endocrinology, Department of Systems Medicine, University of Rome Tor Vergata, Via Montpellier, 1, Rome, 00133 Italy

**Keywords:** Diabetic neuropathy, natural history, progression, regression, risk marker, type 1 diabetes

## Abstract

**Purpose:**

To evaluate the course of diabetic polyneuropathy (DPN) and changes in DPN severity over 5 years in type 1 diabetes (T1D) and the possibility of regression in DPN.

**Methods:**

A retrospective analysis was conducted on 43 participants with T1D (age 38.9 ± 12.5, duration 23.1 ± 13.1 years, 15 males). DPN was diagnosed using symptoms (MNSI-Q ≥ 4) and signs [MDNS ≥ 7 or abnormal vibration (VPT) or thermal perception thresholds (TPT)] and classified as probable when both symptoms and signs were present and possible when only symptoms or signs were observed. Probable DPN was further graded as mild, moderate or severe according to the number of signs identified. Participants were categorized as progressors, regressors, or unchanged based on worsening, improvement or stability of DPN stage.

**Results:**

Participants without DPN decreased from 34.9% to 20.9%, those with possible DPN increased from 37.2% to 41.9%, while probable DPN remained at 37.2%. DPN stage progressed in 37.2%, regressed in 16.3%, and was unchanged in 46.5%. Progressors exhibited higher baseline cholesterol compared to regressors (*P* = 0.027), higher follow-up (FU) HbA1c compared to regressors and unchanged participants (*P* = 0.037), and a lower FU-baseline change (∆) in HDL than both regressors (*P* = 0.029) and unchanged (*P* = 0.013). Multivariate analysis showed that FU HbA1c (OR 1.79, *P* = 0.047) and ∆ HDL (OR 0.88, *P* = 0.030) were predictors of progression, while baseline cholesterol predicted regressors (OR 0.91, *P* = 0.030).

**Conclusions:**

Over 5 years the overall prevalence of DPN increased; however, regression to less severe DPN or complete absence of DPN occurred. These findings indicate that regression of DPN is still possible even in longstanding T1D. Changes in lipids and HbA1c may predict DPN course, suggesting that continued intervention remains beneficial well after a DPN diagnosis.

**Supplementary Information:**

The online version contains supplementary material available at 10.1007/s12020-026-04613-8.

## Introduction

Diabetic polyneuropathy (DPN) affects about 30% of people with diabetes rising to 50% with longer diabetes duration [1, 2]. DCCT/EDIC study reported an increase in prevalence of DPN from 6% at baseline to 33% after 23-year follow-up [3]. While DPN is usually seen as progressive, its natural history remains unclear due to limited knowledge and ongoing debate. Many issues might have hampered a defined comprehension of the natural history of DPN. Multiple pathogenetic mechanisms are recognized whose relevance and time-course action can differ between type 1 (T1D) and type 2 diabetes (T2D). The use of different diagnostic methods, including nerve conduction study (NCS), can influence how progression is identified given that the patterns of deterioration could not be strictly aligned. Moreover, technological and pharmaceutical advances in diabetes care and slow progression of DPN - observed in most recent trials with disease-modifying agents - may hinder comprehension of DPN natural course. Glycaemic control and a multifactorial approach targeted also to weight, blood pressure and lipids are recommended with a different strengths to prevent DPN development and progression [1, 4, 5] Instead, the possibility of DPN regression when diagnostic criteria are met remains underexplored despite its clinical relevance. There is not unanimous acceptance of the dogma of inevitable further deterioration of nerve function as well as of the influencing factors. Few observational longitudinal studies have examined this issue in T2D, and even fewer exist for T1D [6, 7]. Table 1 summarizes the studies discussed below as a background for our research.

**Table 1:** Observational longitudinal studies that evaluate the natural course of diabetic polyneuropathy

Author (year)	Design (setting)	Cohort	FU (years)	Diagnostic criteria	Classification	Baseline prevalence	FU prevalence	Progressors	Regressors	Predictors Progression	Predictors Regression	Comments
T1D												
Ziegler (2021) ^6^	Prospective (German Diabetes Study) (Germany)	179 with T1D (duration *≤* 1 year)	5	• Signs (NDS) • Symptoms (NSS) • NCS Other measures: IENFD, VPT, TPT	• Subclinical • Confirmed asymptomatic • Confirmed symptomatic	12.8%	8.3%	• DPN: 5.8% • NSS: 2.9% • NDS: 5.3% • Peroneal MNCV: 6.2% • Sural SNCV: 6.2% • Malleolar VPT: 6.6%	• DPN: 10.3% • NSS: 4.0% • NDS: 4.1% • Peroneal MNCV: 9.5% • Sural SNCV: 5.1% • Malleolar VPT: 3.0%	None at multivariate analysis	None at multivariate analysis	DPN includes subclinical. Authors’ conclusions: Evidence of early parallel damage to small and large nerve fibres with progressive impairment (lower than in T2D). Some regression to normal occurred in DPN diagnosis (mostly from subclinical DPN) and nerve function tests.
Braffett (2024) ^7^	Prospective (EDIC study) (US)	1324 with T1D (duration 13 years)	26	• MNSI-Q and MNSI-E (annually) • NP (defined as burning and/or allodynia)	• NP DPN+ (NP with MNSI-E > 2)	NP DPN+: 4.0% NP DPN-: 9%	• NP DPN+: 11.0% • NP DPN-: 20%	Cumulative incidence of NP DPN + at EDIC year 26: 36%	Average annual transition from NP DPN + to no NP: 8.7%	↑ HbA1c, age, pulse rate, albuminuria, pulse pressure; smoking; β-blockers; ↓ physical activity; female sex	NA	Authors’ conclusions: The difference between the prevalence and incidence of NP DPN + at FU suggests NP may be transient, often remitting (irrespective of pain drugs). Modifiable risk factors, mainly glucose control, predict NP.
**T2D**												
Määttä (2019) ^8^	Prospective (ADDITION-Denmark) (Denmark) with trial design for 6 years and observation for 7 years	518 with screen-detected T2D	13	• Symptoms (MNSI-Q) at baseline • Symptoms, signs and DPNCheck at 13 years	• No symptoms • Small-fibre symptoms only • Mixed-fibre symptoms • Large-fibre symptoms with/without small-fibre negative symptoms	NA	• No symptoms: 40% • Stable symptoms: 11% • Confirmed DPN: 29%	23% (according to tested hypothesis)	26% (according to tested hypothesis)	NA	NA	Authors’ conclusions: A progressive development from pure small- through mixed- to large-fibre symptoms is not confirmed; symptoms of any categories and course are associated with a higher risk of DPN, being mixed-fibre and large-fibre symptoms stronger predictors.
Ziegler (2021) ^6^	Prospective (German Diabetes Study) (Germany)	291 with T2D (duration *≤* 1 year)	5	• Symptoms (NSS) • Signs (NDS) • NCS Other measures: IENFD, VPT, TPT	• Subclinical • Confirmed asymptomatic • Confirmed symptomatic	14.3%	18.3%	• DPN: 10.4% • NSS: 11.3% • NDS: 15.0% • Peroneal MNCV: 9.8% • Sural SNCV: 8.4% • Malleolar VPT: 18.6% • IENFD: 18.8%	• DPN: 6.5% • NSS: 8.2% • NDS: 11.2% • Peroneal MNCV: 4.6% • Sural SNCV: 3.7% • Malleolar VPT: 5.1% • IENFD: 8.7%	None at multivariate analysis	None at multivariate analysis	DPN includes subclinical. Authors’ conclusions: Evidence of early parallel damage to small and large nerve fibres with progressive impairment (higher than in T1D). Some regression to normal occurred in DPN diagnosis and nerve function tests.
Brask-Thomsen (2024) ^9^	Prospective (Danish Centre for Strategic Research in Type 2 diabetes) (DD2) (Denmark)	184 with T2D (151 at FU) (duration 5.9 years) and 43 controls	5	• Symptoms (MNSI-Q, DN4) • Signs (or bilateral ankle reflexes) • NCS • IENFD	• Subclinical • Possible • Probable • Confirmed	• No DPN: 15.2% • Subclinical: 4.6% • Possible: 17.2% •Probable: 27.2% •Confirmed: 35.8% • Abnormal NCS: 23.8% • Abnormal IENFD: 32.6%	• No DPN: 17.9% • Subclinical: 0.7% • Possible: 16.6% • Probable: 14.6% • Confirmed: 50.3% • Abnormal NCS: 32.5% • Abnormal IENFD: 43.2%	• Any DPN: 23.2% • NCS: 12.6% • IENFD: 16.7% • NCS or IENFD: 21.6%	• Any DPN: 15.2% • NCS: 4.0% • IENFD: 6.1% • NCS or IENFD: 7%	Male sex; baseline and FU ↑ waist and ↑ triglycerides; FU ↑ BMI and ↓ HDL	↓ duration; baseline and FU ↓ HbA1c	Baseline and FU HbA1c were 6.8% and 6.9%. DPN assessment was multilevel. Probable DPN and DPN severity were associated with ↑ risk of confirmed DPN at FU. Authors’ conclusions: Probable and confirmed DPN are associated with progression of DPN signs, whereas only few with possible or no DPN progressed to DPN. Metabolic factors predict the progression, better HbA1c the regression.
Brask-Thomsen (2025) ^10^	Prospective (DD2) (Denmark)	102 with probable / confirmed DPN out of 184 (duration 5.9 years)	5	• Symptoms (MNSI-Q, DN4, NRS) • Signs (or bilateral ankle reflexes) • NCS • IENFD Other measures: TCNS, QST	• Probable • Confirmed • Painful-DPN • Non painful-DPN	• Painful-DPN: 37.3% • Non painful-DPN: 62.8%	• Painful-DPN: 47.1% • Non painful-DPN: 42.2%	• Progression from Non painful-DPN to Painful-DPN: 32.8%	• Transition from Painful-DPN to Non painful-DPN: 23.7% Transition from probable to possible or no DPN: 10.8%	FU ↑ HbA1c; worse baseline NCS and QST	↓ baseline BMI; baseline cholesterol (not multivariate); FU ↓ LDL; worse baseline NCS and QST	Reduction in BMI and waist was protective against pain development in those with Non painful-DPN. Authors’ conclusions: Female sex, worse baseline small- and large-fibre function (NCS and QST) and wider hyposensitivity distribution were associated with pain development while lower baseline BMI, cholesterol, and better small- and large-fibre parameters with relief from pain. Dysesthesia may be a precursor to pain.
Strom (2025) ^11^	Prospective (German Diabetes Study) (Denmark)	52 with T2D (duration *≤* 1 year) and 52 with NGT	5	• Symptoms (NSS) • Signs (NDS) • VPT • TPT • NCS	• Subclinical • Confirmed asymptomatic • Confirmed	• Total: 10.7% (Vs 0%) • Confirmed: 6.4% (Vs 0%)	• Total: 8.5% (Vs 0%) • Confirmed: 6.4% (Vs 0%)	NA (no ↑ in prevalence of Confirmed)	NA (overall ↓ by 48.8% of Subclinical)	NA	NA	Age-related normal reference values were not used. Compared to NGT controls, VPT change was significantly higher in T2D in univariate but not in multivariate analysis. Median annual decline in peroneal MNCV and sural SNCV in NGT was 0.2 m/s and 0.4 m/s.
Strom (2025) ^11^	Prospective (German Diabetes Study) (Denmark)	141 with T2D (duration *≤* 1 year)	10	• Symptoms (NSS) • Signs (NDS) • VPT • TPT • NCS	• Subclinical • Confirmed asymptomatic • Confirmed	• Total: 3.8% • Confirmed: 0%	• Total: 8.6% • Confirmed: 4.8% • Abnormal MNCV: 14.2% • Abnormal SNCV: 30.2%	NA (↑ in prevalence of Confirmed)	NA (transitory ↓ by 50% in prevalence of Subclinical at 5 years)	NA	NA	Age-related normal reference values were not used. Baseline-to-5-year VPT change was related to baseline age. HbA1c was < 7% in 80%, 63% and 52% at baseline, 5 and 10 years. DPN prevalence of DPN was very low at 10-year duration. Authors’ conclusions: In newly diagnosed, well-controlled T2D, nerve function decline is mainly determined by nerve status at diagnosis and age-related changes rather than by disease progression.

Abbreviations: BMI, body mass index; DPN, diabetic polyneuropathy; EDIC, Epidemiology of Diabetes Interventions and Complications; FU, follow-up; HbA1c, glycated haemoglobin; IENFD, intraepidermal nerve fibre density; MNCV, motor nerve conduction velocity; MNSI-E, Michigan Neuropathy Screening Instrument examination; MNSI-Q, MNSI questionnaire; NA, not available; NGT, normal glucose tolerance; NCS, nerve conduction study; NDS, Neuropathy Disability Score; NP, neuropathic pain; NRS, Numerical Rating Scale; NSS, Neuropathy Symptom Score; SNCV, sensory nerve conduction velocity; QST, quantitative sensory testing; T1D, type 1 diabetes; T2D, type 2 diabetes; TCNS, Toronto Clinical Neuropathy Score; TPT, thermal perception threshold; VPT, vibration perception threshold

### Observations in T2D

The ADDITION-Denmark study (518 participants, aged 61 years, with screening-detected T2D) used the Michigan Neuropathy Screening Instrument Questionnaire (MNSI-Q) to classify neuropathic symptoms as small-fibre, mixed-fibre and large-fibre symptoms. The hypothesis that symptoms progress from pure small- to mixed- and finally to large-fibre involvement was not supported by the results, but baseline mixed- and large-fibre symptoms predicted confirmed DPN at 13-year follow-up. Symptom status categories were generally stable although 23% showed a progression and 26% a regression/improvement from one status to another [8] (Table 1).

German Diabetes Study (291 patients with T2D with a duration < 1 year) found DPN (subclinical and confirmed) in 14.3% at baseline and 18.3% at 5 years, with 10.4% progressing to DPN and 6.5% regressing to absence of DPN. No predictors of the course of nerve function tests were identified [6] (Table 1). In the same German Diabetes Study, 52 participants with T2D were matched to those with normal glucose tolerance (NGT) and followed for 5 years, while another 142 subjects with T2D were followed for 10 years [9]. In the first group confirmed DPN prevalence stayed at 6.4% over 5 years with a rate of neurological decline similar to NGT controls, while in the second group confirmed DPN increased from 0% to 4.8% after 10 years. In both groups subclinical DPN decreased by 50% after 5 years [9]. The authors suggested that DPN can be present at diagnosis and that nerve impairment progression is determined by both initial nerve status and physiologic aging, assuming no metabolic deterioration in the first decade [9] (Table 1).

In the Danish Centre for Strategic Research in T2D cohort (DD2) (151 participants with T2D with duration of 5.9 years), confirmed DPN increased from 35.8% to 50.3% after 5 years, but also regression occurred for 9.3% of those with confirmed DPN to probable DPN, for 22.0% of those with probable to possible or no DPN, and for 23.1% of those with possible to no DPN [10]. Overall, 15.2% regressed to a lower DPN status (including 7.3% to no DPN) with 16.7% and 18.6% of those with abnormal regressing to normal NCS and intraepidermal nerve fibre density (IENFD), respectively. Predictors of progression in NCS and IENFD were male sex, a shorter follow-up, higher waist circumference and triglycerides at baseline and follow-up, and higher BMI and lower HDL cholesterol at follow-up, while regression was associated with lower HbA1c at both baseline and follow-up, shorter diabetes duration and a longer follow-up [10] (Table 1). In a subgroup of the DD2 cohort with probable painful or painless DPN, 32.8% progressed from painless to painful and 23.7% changed from painful to painless DPN, being female sex and worse baseline small- and large-fibre function linked to pain development while lower baseline BMI and cholesterol, and better small- and large-fibre measures with pain relief [11].

### Observations in T1D

The EDIC study followed 1,324 individuals with T1D and found at 26-year-follow-up that neuropathic pain with DPN (based on MNSI questionnaire and examination) had a cumulative incidence of 36% and a prevalence of 11%, with annualized remission rates of 8.7%. Key risk factors included higher HbA1c, older age, increased pulse rate, and female sex [7] (Table 1).

German Diabetes Study (179 patients with T1D of < 1 year duration) reported that DPN prevalence dropped from 12.8% at baseline to 8.3% over 5 years with DPN status remaining abnormal in 2.6%, normalizing in 10.3%, and progressing to abnormal in 5.8% [6]. Similarly, other structural and functional nerve measures showed some regression to normal [symptoms in 4%, signs in 4.1%, vibration perception threshold in 3%, and peroneal and sural nerve conduction velocity in 9.5% and 5.1%], but no clear predictors emerged [6] (Table 1).

This overview indicates that neuropathic symptoms are dynamic in both T2D and T1D and that also neuropathic signs and abnormalities in objective measures of nerve structure and function may regress to some extent, though at least in T2D improvement is less frequent than progression.

Thus, we identified two questions of interest: is DPN regression possible in patients with long-standing T1D? and which factors are related to changes in DPN status? Accordingly, this monocentric longitudinal retrospective study was aimed at determining whether a diagnosis of DPN in subjects with T1D is reversible and the predictors of changes in DPN status.

## Materials and methods

### Subjects

This retrospective longitudinal study included individuals with T1D who had undergone at least 2 evaluations of diabetic neuropathy at the outpatient diabetes clinic of the University Hospital of Rome Tor Vergata (Rome, Italy) between 2009 and 2024. Criteria for inclusion were the diagnosis of T1D and age between 18 and 80 years. Individuals with severe comorbidities (i.e., recent cardiovascular events, heart failure, and renal failure [estimated glomerular filtration rate (eGFR) < 45 mL/min/1.73 m^2^]) were excluded, as well as those with peripheral neuropathies from causes other than diabetes, or with advanced peripheral arterial disease requiring revascularization, active limb ulcers, and conditions precluding questionnaires’ comprehension.

The study was approved by the local ethics committee (Lazio Area 2 Ethics Committee, approval no. 91/24) and written informed consent was obtained from all participants.

At baseline and follow-up, we reviewed participants’ clinical and instrumental records, regarding diabetes history, its complications and comorbidities, and the ongoing treatments. Evaluation of macrovascular complications of diabetes included history of acute coronary syndrome, ischemic stroke, and peripheral arterial disease. This latter was also assessed based on the presence of claudication and/or absence of palpable dorsalis pedis and/or posterior tibial pulses. Chronic kidney disease was diagnosed based on the presence of micro- and macroalbuminuria (according to 24-hour albuminuria levels of 30–299 mg/24 h and ≥ 300 mg/24 h, or an albumin-creatinine ratio of 30–299 mg/g and ≥ 300 mg/g, respectively) and/or a reduced eGFR. Recent ophthalmological reports of fundus examinations were considered to establish a diagnosis of diabetic retinopathy, which was classified as background (non-proliferative) or proliferative. Participants were categorized as physically active if they performed regular physical activity for at least 1 h per week, as current smokers if they smoked at least one cigarette per day, and as alcohol consumers if they drank at least one alcoholic beverage per day.

Recent blood tests including HbA1c, serum creatinine, serum cholesterol (total, HDL, LDL), triglycerides, and microalbuminuria were recorded. Anthropometric measurements included height, weight and waist circumferences. Capillary blood glucose levels were measured at the beginning of the neurological examination. Three blood pressure measurements were taken 1 to 2 min apart using an automated device and with participants seated with uncrossed legs.

### Neurological examinations

Neuropathic symptoms were assessed using the MNSI-Q, with abnormal test defined by a cut-off of 4 abnormal items out of 15 [12]. MNSI-Q responses were reviewed by the clinician to resolve any apparent inconsistencies. Neuropathic signs were assessed using the clinical portion of the Michigan Diabetic Neuropathy Score (MDNS) [13], and quantitative sensory testing (QST), which included warm and cold thermal perception thresholds (TPTs) and vibration perception threshold (VPT). VPT was tested at the hallux dorsum and lateral malleolus using the method of limits [14], while warm and cold TPTs were measured at the foot dorsum according to the levels method by means of the Neurosensory Analyzer TSA-II (Medoc, Ramat Yishai, Israel) [15]. Abnormal VPT and TPTs were defined according to age-related reference values, whereas the MDNS was considered abnormal for scores of 7 or higher [13]. In addition, neuropathic pain was identified using *Douleur Neuropathique en 4 Questions* (DN4) screening tool [16]. According to the Toronto consensus criteria, probable DPN was diagnosed in the presence of both neuropathic symptoms and signs, whereas possible DPN defined as the presence of symptoms or signs [1]. Probable DPN was graded as mild, moderate or severe according to the presence of one, two, or three abnormalities among MDNS, VPT and TPTs.

Participants were categorized in three groups - i.e., progressors, regressors and unchanged - based on their change in DPN stage over the follow-up period (Supplementary Fig. 1). Progressors were participants whose DPN stage worsened (e.g., from no DPN to DPN or from possible to probable DPN or from mild to moderate probable DPN), while regressors were participants whose DPN stage improved (e.g., from moderate to mild probable DPN); participants classified as unchanged remained in the same DPN stage.

Gold-standard cardiovascular autonomic reflex tests (CARTs) were used to diagnose cardiovascular autonomic neuropathy (CAN) using DAN computerized data acquisition and analysis system (Microlab Elettronica Sas, Padua, Italy). Standard procedures and age-based reference values were used for deep breathing, lying-to-standing, and the Valsalva manoeuvre tests to get the expiration/Inspiration ratio, 30:15 ratio, and Valsalva ratio. Moreover, an orthostatic hypotension test was performed and a CARTs score generated as the sum of test results (scored as 0 for normal, 1 for borderline, and 2 for abnormal; range 0–8). Early CAN was diagnosed if one CART was abnormal, while confirmed CAN with at least two abnormal CARTs [17].

### Statistical analysis

The distribution of data was assessed using the Shapiro-Wilk test. Normally distributed variables were expressed as mean ± standard deviation (SD), whereas non-normally distributed variables as median (interquartile range [IQR]). Descriptive statistics were applied to characterize participants’ clinical variables. The unpaired Student’s t-test and the Mann-Whitney test were used to compare continuous variables between two groups, according to data distribution. Comparisons among more than two groups were performed using one-way ANOVA or Kruskal-Wallis test for normally or non-normally distributed variables. Post hoc pairwise comparisons were carried out using Tukey’s test after ANOVA and Dunn’s test after Kruskal-Wallis, with the latter adjusted according to Holm correction.

Changes over time in non-normally distributed continuous variables were assessed using the Wilcoxon signed-rank test, while McNemar’s test was applied to evaluate changes in the proportion of participants with abnormal neurologic tests. Correlations between variables were analysed using Pearson’s correlation coefficient (r) for normally distributed and Spearman’s rank correlation coefficient (rho) for non-normally distributed data. To evaluate the predictive value of clinical variables for the progression or regression of DPN, a multivariate logistic regression analysis was performed adjusting for sex, age, BMI, and diabetes duration.

All statistical analyses were done using Statistics/Data Analysis (STATA) (StataCorp LP, TX, USA). A two-tailed value of *P* < 0.05 was considered significant.

## Results

The participants’ mean age and diabetes duration at baseline were 38.9 ± 12.4 and 23.1 ± 13.1 years, and their median (IQR) HbA1c was 7.8% (1.5%) (Supplementary Table 1).

**Fig. 1:**
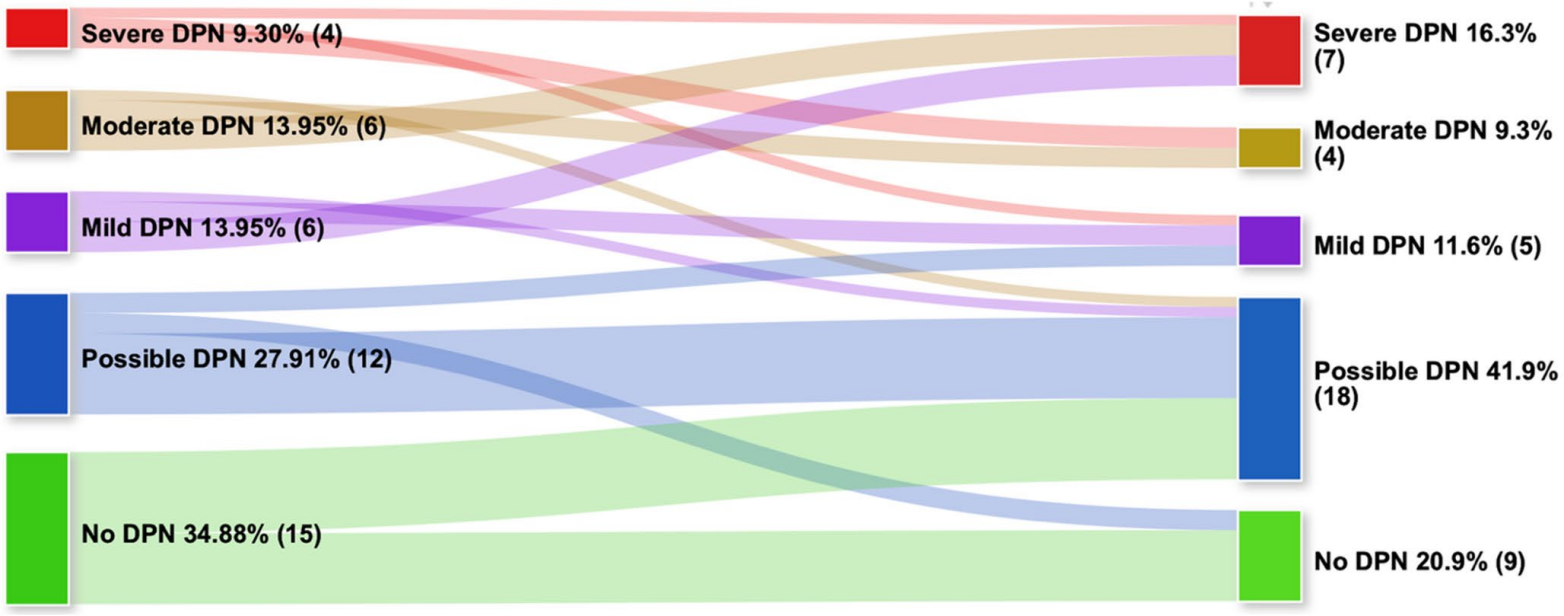
Sankey diagram with the changes in DPN status from baseline to follow-up

At baseline, 15 participants (34.9%) were not diagnosed with DPN (noDPN), 12 (27.9%) were diagnosed with possible DPN, and 16 (37.2%) with probable DPN. Among those with probable DPN, 6 participants (13.95%) had mild DPN, 6 (13.95%) had moderate DPN, and 4 (9.30%) had severe DPN. At follow-up, the prevalence of noDPN fell to 20.9%, possible DPN increased to 41.9%, while probable DPN remained stable at 37.2%. Thus, DPN progressed in 16 participants (37.2%), regressed in 7 (16.3%), and was unchanged in 20 (46.5%) (Fig. 1).

Regarding baseline characteristics according to DPN stage (Table 2), total cholesterol tended to be higher in participants without DPN compared to those with possible DPN (*P* = 0.057), which was consistent with the higher rate of statin use observed in participants with possible DPN (*P* = 0.028). Moreover, the prevalence of diabetic retinopathy was higher among participants with probable DPN compared to those with possible DPN or without DPN (*P* = 0.036 and *P* = 0.025, respectively).

**Table 2:** Clinical and neurological characteristics at baseline according to DPN status

Patients	No DPN	Possible DPN	Probable DPN	*P* No Vs Possible	*P* No Vs Probable	*P* Possible Vs Probable
Number	15	12	16	---	---	---
Sex (M: F)	6:9	4:8	5:11	0.999	0.716	0.999
Age (years)	38.13 ± 10.59	37.75 ± 17.69	40.50 ± 9.82	0.945	0.524	0.604
Diabetes duration (years)	22.29 ± 12.94	23.17 ± 18.65	23.88 ± 8.11	0.886	0.683	0.893
BMI (Kg/m^2^)	23.54 ± 2.86	23.36 ± 2.97	25.01 ± 3.17	0.871	0.187	0.173
HbA1c (%)	7.90 (0.85)	7.25 (1.48)	8.00 (1.00)	0.383	0.529	0.189
HbA1c (mmol/mol)	63.00 (9.00)	54.50 (15.50)	64.00 (11.00)	0.368	0.561	0.199
eGFR (ml/min/1.73 m^2^)	102.98 ± 18.38	103.22 ± 22.27	93.15 ± 22.61	0.978	0.218	0.306
Total cholesterol (mg/dl)	170.75 ± 26.61	141.25 ± 27.45	163.39 ± 26.82	**0.028**	0.498	0.084
HDL cholesterol (mg/dl)	55.42 ± 15.07	52.00 ± 7.33	55.62 ± 14.46	0.561	0.973	0.522
Triglycerides (mg/dl)	74.50 (57.50)	62.50 (5.75)	79.00 (43.00)	0.666	0.767	0.637
Statins (%)	0.00	33.33	25.00	**0.028**	0.101	0.691
Systolic BP (mmHg)	111.67 ± 15.77	118.75 ± 14.16	117.40 ± 18.99	0.237	0.376	0.840
Diastolic BP (mmHg)	67.00 ± 9.22	70.42 ± 6.90	68.20 ± 7.98	0.297	0.706	0.454
Retinopathy (%)	35.71	33.33	80.00	0.999	**0.025**	**0.036**
Nephropathy (%)	0.00	33.33	21.43	0.054	0.222	0.643
Hypertension (%)	26.66	33.33	35.71	1.000	0.700	1.000
Cardiovascular disease (%)	0.00	16.66	6.25	0.188	1.000	0.560
Smokers (%)	33.33	16.66	43.75	0.408	0.716	0.223
Physical activity (%)	50.00	41.66	31.25	0.713	0.457	0.698
Alcohol (%)	21.43	25.00	20.00	1.000	1.000	1.000
MNSI-Q	1.00 (1.00)	2.00 (3.25)	6.50 (3.25)	0.371	**< 0.001**	**< 0.001**
MDNS	2.00 (2.50)	2.00 (4.25)	6.00 (6.50)	0.443	**0.003**	**0.037**
VPT hallux (Volt)	8.58 (2.08)	10.65 (4.39)	21.49 (13.16)	0.231	**0.004**	0.231
VPT lateral malleolus (Volt)	9.20 (3.23)	12.93 (6.64)	23.81 (15.42)	0.201	**0.004**	0.201
Cold thermal threshold (C°)	30.63 (0.96)	31.30 (2.08)	24.88 (9.78)	0.951	**0.003**	**0.003**
Warm thermal threshold (C°)	33.65 (3.28)	33.20 (2.30)	39.45 (6.54)	0.890	**0.004**	**0.004**
DN4	0.00 (0.00)	0.00 (1.50)	3.00 (3.25)	0.233	**< 0.001**	**0.008**
CARTs score	0.00 (0.00)	1.00 (2.00)	4.50 (5.50)	0.079	**< 0.001**	**0.027**
Expiration-Inspiration ratio	1.43 (0.28)	1.30 (0.23)	1.12 (0.15)	0.154	**< 0.001**	0.105
30/15 ratio	1.29 (0.29)	1.18 (0.16)	1.07 (0.18)	0.099	**0.007**	0.375
Valsalva ratio	1.80 ± 0.35	1.72 ± 0.31	1.36 ± 0.32	0.802	**0.006**	**0.040**
Orthostatic hypotension (mmHg)	10.00 (5.00)	10.00 (1.25)	15.00 (20.00)	0.894	0.486	0.486
With CAN (early and confirmed) (%)	0.00	41.66	68.75	**0.010**	**< 0.001**	0.249
With confirmed CAN (%)	0.00	8.33	56.25	0.444	**< 0.001**	**0.016**

Group comparisons: ANOVA with Tukey post-hoc test (normally distributed data); Kruskal-Wallis test with Dunn’s post-hoc test and Holm correction for multiple comparisons (non-normally distributed data)

Abbreviations: BMI, body mass index; BP, blood pressure; CAN, cardiovascular autonomic neuropathy; CART, cardiovascular autonomic reflex test; DN4, Douleur Neuropathique en 4 questions; DPN, diabetic polyneuropathy; eGFR, estimated glomerular filtration rate; F, female; HbA1c, glycated haemoglobin; HDL, high-density lipoprotein; M, male; MDNS, Michigan Diabetic Neuropathy Score; MNSI-Q, Michigan Neuropathy Screening Instrument Questionnaire; VPT, Vibration perception threshold

Values are presented as number (%), and as mean ± standard deviation or median (interquartile range) according to their normal or non-normal distribution. Statistically significant P-values (*p* < 0.05) are shown in bold

As expected, at baseline, all the neurological parameters differed significantly between participants without DPN and those with probable DPN with some differences between possible DPN and probable DPN. Participants with probable DPN showed a higher CARTs score compared to those without DPN and those with possible DPN (*P* < 0.001 and *P* = 0.027, respectively), as well as a lower Valsalva ratio (*P* = 0.006 and *P* = 0.040, respectively). Moreover, participants with probable DPN had a lower expiration/inspiration ratio and 30/15 ratio compared to those without DPN (*P* < 0.001 and *P* = 0.007, respectively). Overall, a significantly higher prevalence of confirmed CAN was observed in participants with probable DPN compared to those with possible DPN or no DPN (*P* = 0.016 and *P* < 0.001, respectively).

**Fig. 2:**
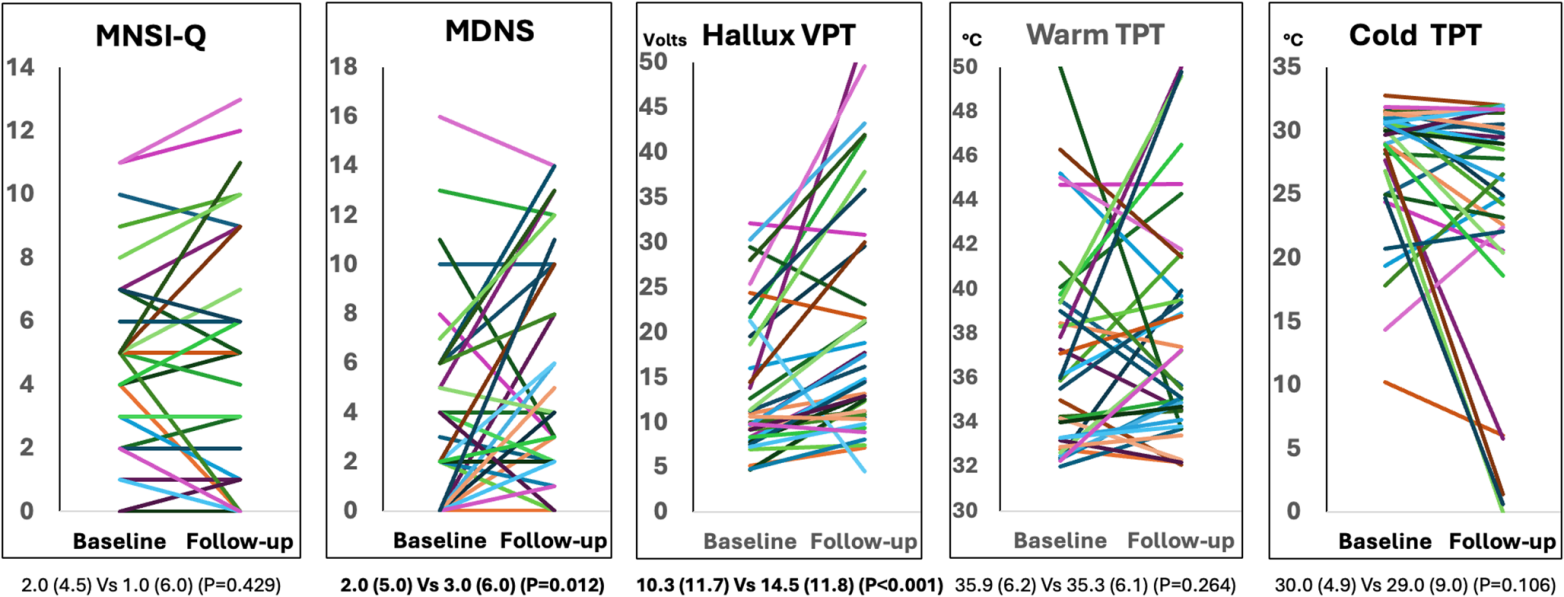
Changes in DPN measures from baseline to follow-up. Wilcoxon signed-rank test was used to assess the differences between median values of baseline and follow-up. Percentage of abnormal MNSI-Q went from 44.2% to 39.5% (*P* = 0.500), that of abnormal MDNS from 13.9% to 30.2% (*P* = 0.065), that of abnormal VPT from 51.3% to 64.1% (*P* = 0.227), and that of abnormal TPT from 35.1% to 53.8% (*P* = 0.039)

When considering the course of neurological measures over follow-up, VPT (*P* < 0.001) and MDNS (*P* = 0.012) were the only showing significant deterioration, whereas the warm and cold TPT, and the MNSI-Q did not exhibit significant changes (*P* > 0.05) (Fig. 2). However, the rate of abnormalities in warm and/or cold TPTs significantly rose from 35.1% at baseline to 53.8% at follow-up (*P* = 0.039). No differences in both median values [0.50 (2.00) Vs 1.00 (2.50), *P* = 0.533] and in percentage of abnormal values (≥ 4) were observed for DN4 from baseline to follow-up (16.3% to 20.9%, *P* = 0.479).

**Fig. 3:**
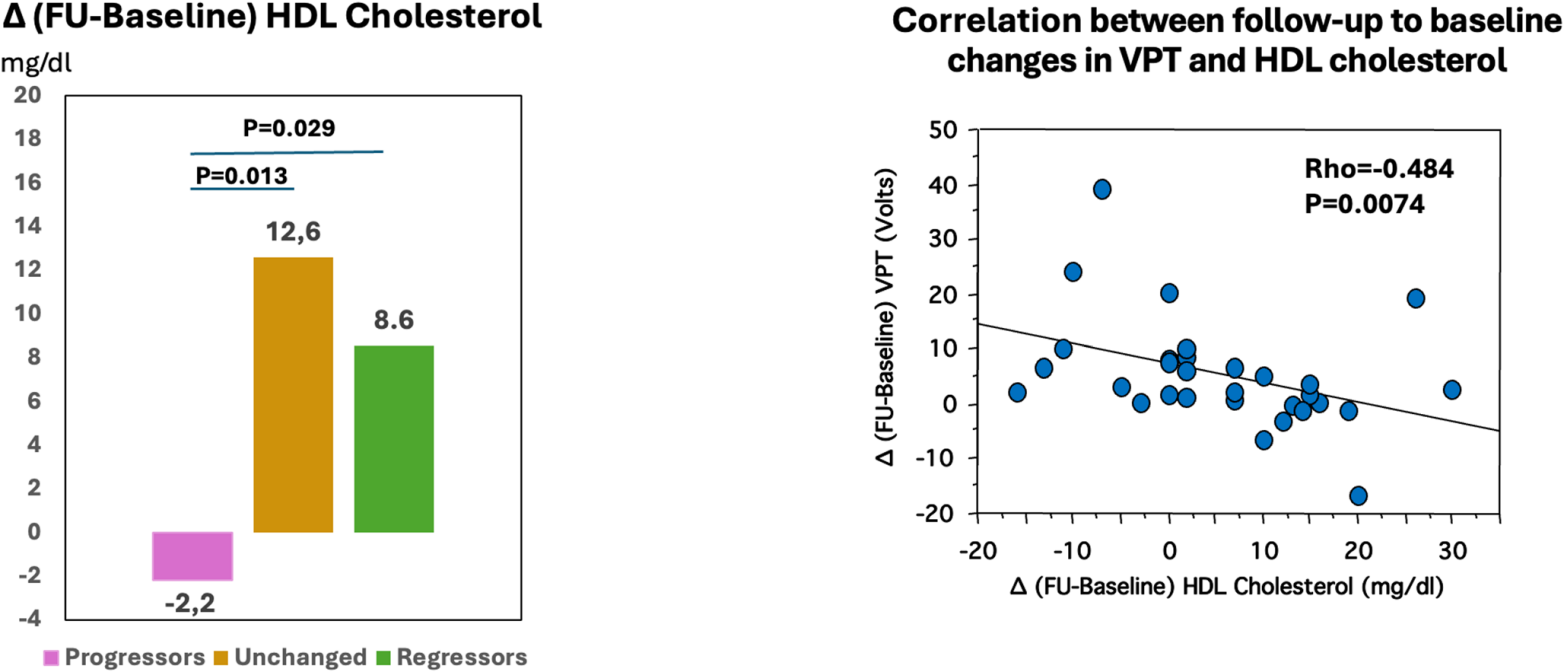
Change (∆) in HDL Cholesterol from follow-up to baseline according to DPN course and its correlation with ∆ VPT

Progressors showed higher baseline cholesterol than regressors (*P* = 0.027) (Table 3; Fig. 3), and higher follow-up HbA1c than regressors plus those with unchanged stage [8.00 (1.40) Vs 7.05 (1.93), *P* = 0.037] (Table 4). In addition, at baseline the prevalence of smokers was unexpectedly higher among regressors than progressors (*P* = 0.045) (Table 3).

**Table 3:** Clinical and neurological characteristics at baseline according to changes in DPN status

Baseline						
Variable	Progressors (P) N. 16	Unchanged (U) N. 20	Regressors (R) N. 7	P P vs. U	P P vs. R	P U vs. R
Sex (M: F)	7:9	6:14	2:5	0.493	0.657	1.000
Age (years)	36.38 ± 10.77	40.10 ± 13.58	41.29 ± 13.59	0.378	0.363	0.844
Duration (years)	24.38 ± 13.75	21.62 ± 13.35	24.57 ± 12.07	0.547	0.974	0.610
BMI (kg/m²)	24.49 ± 3.21	23.55 ± 2.99	24.38 ± 2.97	0.371	0.938	0.534
Follow-up (years)	5.05 (3.64)	5.50 (3.56)	3.75 (1.29)	0.987	0.237	0.237
HbA1c (%)	8.20 (2.00)	7.60 (0.70)	7.80 (1.33)	0.613	0.585	1.000
HbA1c (mmol/mol)	66.00 (21.00)	59.50 (8.00)	61.00 (15.50)	0.613	0.585	1.000
eGFR (ml/min/1.73 m^2^)	105.55 ± 26.03	99.95 ± 19.23	90.43 ± 12.85	0.939	0.485	0.611
Total cholesterol (mg/dl)	167.33 ± 19.48	163.56 ± 33.16	142.17 ± 18.43	0.756	**0.027**	0.150
HDL cholesterol (mg/dl)	56.00 ± 16.34	56.00 ± 11.64	50.50 ± 12.72	0.912	0.506	0.393
Triglycerides (mg/dl)	81.00 (98.00)	64.50 (32.50)	66.00 (10.50)	1.000	1.000	1.000
Statins (%)	12.50	20.00	28.57	0.672	0.557	0.633
Systolic BP (mmHg)	118.67 ± 15.52	111.25 ± 15.88	122.29 ± 18.83	0.384	0.878	0.281
Diastolic BP (mmHg)	70.00 ± 7.32	68.25 ± 9.07	65.43 ± 6.68	0.805	0.444	0.712
Retinopathy (%)	57.14	33.33	71.43	0.283	0.656	0.177
Nephropathy (%)	14.29	18.75	16.66	1.000	1.000	1.000
Hypertension (%)	37.50	30.00	42.86	0.729	1.000	0.653
Cardiovascular disease (%)	6.25	5.00	14.29	1.000	0.526	0.459
Smokers (%)	12.50	40.00	57.14	0.133	**0.045**	0.662
Physical activity (%)	40.00	40.00	42.86	1.000	1.000	1.000
Alcohol (%)	25.00	15.79	28.57	0.677	1.000	0.588
MNSI-Q	3.50 (4.25)	1.00 (3.25)	5.00 (4.50)	0.159	0.159	**0.014**
MDNS	2.00 (3.50)	2.00 (4.00)	6.00 (8.00)	0.764	0.764	0.698
VPT hallux (Volt)	9.70 (6.38)	10.33 (10.09)	21.33 (18.75)	0.662	0.402	0.662
VPT lateral malleolus (Volt)	13.30 (6.77)	12.18 (12.47)	24.16 (16.45)	0.815	0.517	0.815
Cold thermal threshold (C°)	30.17 (2.85)	30.00 (2.50)	24.70 (10.50)	0.932	0.932	0.932
Warm thermal threshold (C°)	36.70 (4.69)	34.20 (3.90)	40.35 (9.35)	0.896	0.896	0.616
DN4	1.00 (2.00)	0.00 (1.50)	2.00 (3.50)	0.421	0.175	0.054

Group comparisons: ANOVA with Tukey post-hoc test (normally distributed data); Kruskal-Wallis test with Dunn post-hoc test and Holm correction for multiple comparisons (non-normally distributed data)

Abbreviations: BMI, body mass index; BP, blood pressure; DN4, Douleur Neuropathique en 4 questions; HbA1c, glycated hemoglobin; MDNS, Michigan Diabetic Neuropathy Score; MNSI-Q, Michigan Neuropathy Screening Instrument-Questionnaire; VPT, Vibration perception threshold

Values are presented as number (%), and as mean ± standard deviation or median (interquartile range) according to their normal or non-normal distribution. Statistically significant P-values (*p* < 0.05) are shown in bold

**Table 4:** Clinical and neurological characteristics at follow-up according to changes in DPN status

Follow-up						
Variable	Progressors (P) N. 16	Unchanged (U) N. 20	Regressors (R) N. 7	P P vs. U	P P vs. R	P U vs. R
Sex (M: F)	7:9	6:14	2:5	0.493	0.657	1.000
Age (years)	42.81 ± 10.82	45.55 ± 14.08	45.57 ± 12.74	0.799	0.882	1.000
Duration (years)	30.75 ± 13.59	27.10 ± 14.24	29.14 ± 10.62	0.702	0.963	0.937
BMI (kg/m²)	25.09 ± 4.34	24.25 ± 3.36	23.63 ± 2.85	0.781	0.661	0.921
Follow-up (years)	5.05 (3.64)	5.50 (3.56)	3.75 (1.29)	0.987	0.237	0.237
HbA1c (%)	8.00 (1.40)	6.80 (1.78)	7.80 (1.40)	0.056	0.615	0.476
HbA1c (mmol/mol)	64.00 (15.50)	51.00 (19.75)	61.50 (15.75)	0.056	0.615	0.476
eGFR (ml/min/1.73 m^2^)	99.29 ± 21.91	94.76 ± 15.87	108.92 ± 16.93	0.748	0.530	0.241
Total cholesterol (mg/dl)	173.00 (23.00)	175.00 (29.00)	169.50 (24.50)	1.000	1.000	1.000
HDL cholesterol (mg/dl)	56.88 ± 13.17	64.30 ± 13.42	58.17 ± 16.20	0.252	0.979	0.606
Triglycerides (mg/dl)	77.00 (38.00)	82.00 (33.00)	62.00 (12.75)	1.000	0.503	0.503
Systolic BP (mmHg)	119.69 ± 15.73	124.20 ± 14.60	119.83 ± 26.32	0.710	1.000	0.846
Diastolic BP (mmHg)	72.25 ± 12.52	73.50 ± 7.98	71.33 ± 12.57	0.934	0.982	0.899
Retinopathy (%)	68.75	45.00	57.14	0.191	0.657	0.678
Nephropathy (%)	26.66	5.00	14.29	0.141	1.000	0.459
Hypertension (%)	43.75	40.00	71.43	1.000	0.371	0.209
Cardiovascular disease (%)	12.50	5.00	28.57	0.574	0.557	0.156
Smokers (%)	31.25	30.00	66.66	1.000	0.178	0.163
Physical activity (%)	37.50	31.58	33.33	0.736	1.000	1.000
Alcohol (%)	12.50	15.00	16.66	1.000	1.000	1.000
MNSI-Q	4.00 (6.75)	1.00 (3.25)	5.00 (7.50)	0.479	0.892	0.892
MDNS	3.50 (9.25)	3.00 (3.25)	3.00 (5.00)	1.000	1.000	1.000
VPT hallux (Volt)	17.58 (17.38)	13.05 (10.19)	9.88 (12.44)	0.540	0.221	0.540
VPT lateral malleolus (Volt)	21.50 (20.14)	15.43 (16.67)	15.60 (21.31)	0.760	0.760	0.912
Cold thermal threshold (C°)	24.38 (18.28)	30.35 (6.83)	29.70 (5.95)	0.111	0.612	0.612
Warm thermal threshold (C°)	37.30 (7.84)	34.98 (5.99)	35.50 (2.80)	1.000	1.000	1.000
DN4	1.00 (2.25)	0.00 (1.00)	3.00 (4.50)	0.323	0.423	0.174

Group comparisons: ANOVA with Tukey post-hoc test (normally distributed data); Kruskal-Wallis test with Dunn’s post-hoc test and Holm correction for multiple comparisons (non-normally distributed data)

Abbreviations: BMI, body mass index; BP, blood pressure; DN4, Douleur Neuropathique en 4 questions; HbA1c, glycated hemoglobin; MDNS, Michigan Diabetic Neuropathy Score; MNSI-Q, Michigan Neuropathy Screening Instrument-Questionnaire; VPT, Vibration perception threshold

Values are presented as number (%), and as mean ± standard deviation or median (interquartile range) according to their normal or non-normal distribution. Statistically significant P-values (*p* < 0.05) are shown in bold

Moreover, progressors had lower follow-up-to-baseline change (∆) in HDL (-2.2±12.0) compared to regressors (12.6±8.1; *P* = 0.029) and those with unchanged stage (8.6±9.1; *P* = 0.013) (Fig. 3). Supplementary Table 2 shows the ∆ of all the clinical variables and DPN measures. Furthermore, follow-up-to-baseline ∆ in VPT were negatively correlated with follow-up-to-baseline ∆ in HDL cholesterol (rho=-0.484, *P* = 0.0074), highlighting that a greater decrease in HDL over time was linked to a greater impairment in VPT (Fig. 3).

In a multivariate analysis adjusted for age, sex, BMI and diabetes duration, follow-up HbA1c (%) (OR = 1.793; 95% CI: 1.008–3.188; *P* = 0.047) and follow-up-to-baseline ∆ in HDL (OR = 0.884; 95% CI: 0.791–0.988; *P* = 0.030) were independent predictors of progression, while baseline total cholesterol predicted regression (OR = 0.912; 95% CI: 0.839–0.991; *P* = 0.030) (Table 5).

**Table 5:** Multivariate logistic regression analyses for the condition of Progressor and Regressor (adjusted for sex, age, BMI, and duration)

	*R* ^2^	Odds ratio	Odds ratio 95% CI	*P*-value
Progressor				
Sex (male)	0.19	1.968	0.358–10.803	0.436
Age (year)	0.19	0.908	0.819–1.007	0.068
BMI (Kg/m^2^)	0.19	1.042	0.784–1.385	0.777
Diabetes duration (year)	0.19	1.086	0.992–1.189	0.074
FU HbA1c (%)	0.19	1.793	1.008–3.188	**0.047**
	***R*** ^2^	**Odds ratio**	**Odds ratio 95% CI**	***P*** **-value**
Progressor				
Sex (male)	0.26	2.923	0.384–22.275	0.301
Age (year)	0.26	0.967	0.849–1.102	0.613
BMI (Kg/m^2^)	0.26	0.939	0.665–1.326	0.722
Diabetes duration (year)	0.26	1.018	0.915–1.132	0.743
Δ (FU-Baseline) HDL (mg/dl)	0.26	0.884	0.791—0.988	**0.030**
	***R*** ^2^	**Odds ratio**	**Odds ratio 95% CI**	***P*** **-value**
Regressor				
Sex (male)	0.32	0.060	0.003–1.405	0.080
Age (year)	0.32	1.034	0.937–1.140	0.511
BMI (Kg/m^2^)	0.32	1.658	0.938–2.931	0.082
Diabetes duration (year)	0.32	0.979	0.893–1.072	0.642
Baseline cholesterol (mg/dl)	0.32	0.912	0.839–0.991	**0.030**

Abbreviations: BMI, body mass index; HbA1c, glycated hemoglobin; CI, confidence interval; Δ HDL, difference between follow-up and baseline HDL cholesterol; FU, Follow-up. Statistically significant P-values (*p* < 0.05) are shown in bold

## Discussion

Data on the natural history of DPN is still limited in particular in T1D. The possible regression of DPN also in a clinical stage is still under debate and the dogma of DPN irreversibility seems to be undermined by some recent observations in both T1D and T2D.

In recent longitudinal studies in T2D, around 7% of participants regressed from DPN to no DPN at 5-year-follow-up with percentages of regressors from abnormality to normality of objective measures of DPN ranging from 3.7% to 6.1% [6, 10] and of neuropathic symptoms until 26% at 13-year follow-up [8]. In T1D, regression from DPN diagnosis to no DPN was described in 10.3% after 5-year-follow-up together with normalization of both symptoms and various DPN measures in proportions of patients from 4% to 9.5% [6]. An even greater variability of the course of painful DPN (with a diagnosis based on MNSI) was described in the EDIC study with an annualized remission rate of 8.7% [7].

Thus, this study aimed at addressing the question whether DPN regression is possible in patients with long-lasting T1D and which factors are related to changes in DPN status.

In this single-centre longitudinal study in young adults with long-lasting T1D, at 5-year-follow-up we found the prevalence of noDPN decreased (from 35% to 21%), that of possible DPN increased (from 28% to 42%), while probable DPN remained stable at 37%. At follow-up, 47% remained in their original DPN status, while 37% showed progression and 16% regression. VPT exhibited a significant deterioration over the follow-up period [from 10.33 (11.74) to 14.55 (11.78) Volts] and follow-up-to-baseline changes in VPT and HDL were inversely correlated. Higher follow-up HbA1c and follow-up-to-baseline decrease in HDL were independent predictors of DPN progression, while lower baseline total cholesterol predicted DPN regression.

### Heterogeneous course of DPN

This study documents an overall progression of DPN with an increase in the prevalence of possible DPN, a significant deterioration of hallux VPT and a higher prevalence of abnormal TPT. However, there was a small proportion of participants (16%) who improved their DPN status changing from a higher to a lower degree of probable DPN or from probable to possible DPN or even from possible to noDPN.

These results align with data on DPN regression from the cited German study in T1D [6] and Danish study in T2D [10]. The percentage of regressors was more similar to the T2D group (15.2%) [10] than the T1D group (10.3%) [6], despite incomplete overlapping in DPN staging criteria.

### How to interpret these findings?

A critical point to disentangle the meaning of these findings concerns the criteria used to determine either progression or regression of DPN [18].

We defined the progression and regression according to the change in DPN status. The definition of DPN status considered the Toronto Consensus criteria [1], i.e., the presence of symptoms or signs for possible DPN and of both for probable DPN and was based on the abnormality of MNSI-Q, clinical portion of MDNS, VPT and TPT with a further grading of severity of probable DPN according to the number of abnormalities among MDNS, VPT and TPT. Thus, the concept of progression and regression considered changes in both diagnostic level (as from possible DPN to noDPN) and severity (as from severe probable DPN to mild probable DPN).

Another point relates to the validity of test outcomes. Standardization and reproducibility of diagnostic tests are essential to identify changes over time [19] and to limit the eventuality that the observed changes are in part dependent on the variability in test results. We used validated measures to assess and score neuropathic symptoms and signs [12, 13]. MNSI-Q includes both positive and negative sensory symptoms, it has a sensitivity and specificity at the cut-off of 4 of 40% and 92% for confirmed DPN [12] and has been widely used in clinical studies in T1D [20]. The clinical portion of MDNS explores vibration, pinprick and touch pressure perception, muscle strength and reflexes of upper and lower limbs and at the cut-off of 7 has a sensitivity and specificity of 64.1% and 81.0% compared to nerve conduction studies [19]. These tools were used according to the instructions provided by the authors [13]. Moreover, validated modalities for both VPT and TPT were used according to standard procedures and age-related reference normative values [14, 15, 21]. The DPN assessment was done in both the occasions by expert personnel confident with neurological evaluation and different instrumental modalities.

### Course of neuropathic symptoms and signs

Neuropathic symptoms might be more susceptible to changes over time than signs. Although painful symptoms of painful DPN can last for many years despite pain treatment, a spontaneous remission of neuropathic symptoms of painful DPN over time is reported in a remarkable percentage of people with diabetes, i.e. 23.3% [22] and 28.9% [11] as well as the possibility of transient and remitting neuropathic pain associated with DPN in T1D [7]. We did not observe a significant decrease in MNSI-Q score [from 2.00 (4.50) to 1.00 (6.00)] or in the percentage of MNSI-Q abnormalities (from 44.2% to 39.5%, *P* = 0.500) at 5-year-follow-up and we cannot consider that the remission is due to a net or prevalent decrease in neuropathic symptoms. However, a deterioration of objective measures was more evident as for median VPT and percentage of abnormalities of TPT in addition to a significant increase in median MDNS (*P* = 0.012) and non-significant in the percentage of abnormal MDNS (from 13.9% to 30.2%, *P* = 0.065). Thus, in adults with long-standing T1D we observed an overall declining trend in objective measures that nonetheless does not exclude a number of cases showing improvement in neurological measures and DPN status (Figs. 1 and 2). The pathophysiological nature of DPN regression in both signs and symptoms is beyond the scope of this study but may involve a more dynamic course of degeneration and regeneration processes than previously thought.

### Clinical predictors of regression and progression

We found that changes in HDL from follow-up to baseline related to VPT deterioration and were independent predictors of DPN progression, while total cholesterol at baseline predicted DPN regression. Moreover, higher follow-up HbA1c independently predicted DPN progression. Both HbA1c and dyslipidemia are recognized risk markers of DPN in T1D [20, 23]. The pathogenetic role of glycaemic control is well established in T1D as well as the beneficial effects of targeting glycaemic control [24]. On the other hand, there is no clear evidence of preventive efficacy of targeting lipids, despite dyslipidemia being increasingly considered a pathogenetic factor of nerve damage through the effects on advanced glycation end-products, proinflammatory action of oxidized LDL, and the excess of non-esterified fatty acids leading to bioenergetic failure, increased reactive oxygen species production, and mitochondrial damage [25]. Confirming previous observations in T1D [7] and T2D [10], this study suggests that targeting glycaemic control and dyslipidemia might be of potential benefit also when DPN has already developed at least at the individual level in T1D. However, ad hoc designed longitudinal studies are needed to establish this.

Baseline smoking was associated with DPN regression in contrast to the recognized role of smoking as a risk factor for DPN and unlike what shown for painful DPN in the EDIC study [7]. Changes in smoking habit during the follow-up (no difference in smoking at follow-up) might mitigate this unexpected finding.

### Strengths and limitations

This is a well-characterized population regarding both clinical variables and a standardized multilevel diagnostic assessment of DPN together with the use of gold-standard methods for CAN diagnosis.

We recognized as limitations of the study the small sample size, its being a single-centre study, the retrospective design and the lack of nerve conduction studies. Moreover, continuous glucose monitoring data were not collected at the time of neurological examinations and are currently unavailable for a sufficient number of participants to permit statistical analysis.

In conclusion, after 5 years, we observed an increase in overall DPN prevalence in a cohort with long-standing T1D, mainly driven by possible DPN diagnoses, with a deterioration slower than expected. Although progression was more common, some cases regressed to less severe DPN or even noDPN, reflecting a dynamic condition amenable to continued intervention. We found lipids and HbA1c and their changes as predictors of DPN course. This suggests that in T1D lipids, beyond glycaemic control, may modify the natural course of DPN and strengthens the role of intervention in the early stages of DPN as well as the need for early screening of DPN. Larger studies are needed to support this view.

## Summary

### What is known about this research topic?

- The natural course of diabetic polyneuropathy (DPN) is poorly investigated and the dogma of DPN irreversibility is now being questioned by studies in type 2 diabetes.
- One study has shown regression of initial nerve alterations in newly diagnosed type 1 diabetes.

### What this study adds and its future implications

- Even in long-standing type 1 diabetes, both DPN progression and regression can occur.
- Lipids and HbA1c changes play a role as predictors of different DPN trajectories.
- The results support continued intervention even long after DPN diagnosis and a tailored approach. Identifying biomarkers for different DPN paths is a valuable goal for larger studies.

## Supplementary Information

Below is the link to the electronic supplementary material.

Supplementary Material 1

## Data Availability

The data generated and analysed during the current study are available from the corresponding author on a reasonable request.

## Declarations

### Prior Presentation

Part of this study was presented at the 35th Annual Meeting of NEUROdiab - Diabetic Neuropathy Study Group in Bucharest (Romania), 14–18 September 2025.
