# Supplementary material for: Heterogeneous course of diabetic polyneuropathy in type 1 diabetes over 5 years: is regression possible?
Source: Endocrine. 2026 Apr 27;91(1):147. doi: 10.1007/s12020-026-04613-8 (PMC13121219; doi:10.1007/s12020-026-04613-8)

Heterogeneous course of diabetic polyneuropathy in type 1 diabetes over 5 years: is regression possible?

Pietro Pertile, Ilenia D'Ippolito, Sara Mascambroni, Cinzia D'Amato, Aikaterini Andreadi, Davide Lauro, Vincenza Spallone

Corresponding author

Vincenza Spallone, MD, PhD, University of Rome Tor Vergata, Endocrinology, Department of Systems Medicine, Via Montpellier, 1, 00133 Rome, Italy, e-mail: vispa2@gmail.com, vincenza.spallone@uniroma2.it

https://orcid.org/0000-0002-8905-216X

Supplementary Table 1. Baseline demographic, clinical and neurologic characteristics of participants with type 1 diabetes.

Characteristics	
Number	43
Sex (M:F)	15 : 28
Age (years)	38.91 ± 12.46
Diabetes duration (years)	23.12 ± 13.07
BMI (kg/m^2)	24.04 ± 3.03
HbA1c (%)	7.83 (1.45)
HbA1c mmol/mol)	62.00 (17.50)
eGFR (ml/min/1.73 m^2)	99.32 ± 20.99
Total cholesterol (mg/dl)	160.70 ± 28.52
HDL cholesterol (mg/dl)	54.67 ± 13.06
Triglycerides (mg/dl)	66.00 (39.00)
Systolic BP (mmHg)	115.74 ± 16.48
Diastolic BP (mmHg)	70.00 (15.00)
Retinopathy (%)	46.51
Nephropathy (%)	11.63
Hypertension (%)	34.88
Cardiovascular disease (%)	6.98
Smokers (%)	32.56
Physical activity (%)	39.53
Alcohol (%)	20.93
MNSI-Q	2.00 (4.50)
MDNS	2.00 (5.00)
VPT hallux (Volt)	10.33 (12.27)
VPT lateral malleolus (Volt)	12.98 (13.82)
Cold thermal threshold (°C)	30.00 (4.94)
Warm thermal threshold (°C)	35.50 (5.98)
DN4	0.50 (2.00)
Without DPN (%)	34.88
With possible DPN (%)	27.91
With probable DPN (%)	37.21
With mild probable DPN (%)	13.95
With moderate probable DPN (%)	13.95
With severe probable DPN (%)	9.30
CARTs score	1.00 (3.50)
Expiration-Inspiration ratio	1.30 (0.34)
30/15 ratio	1.18 (0.23)
Valsalva ratio	1.63 ± 0.37
Orthostatic hypotension (mmHg)	10.00 (5.00)
With CAN (early and confirmed) (%)	37.21
With confirmed CAN (%)	23.26
Follow-up (years)	4.92 (3.75)

Abbreviations: BMI, body mass index; BP, blood pressure; CAN, cardiovascular autonomic neuropathy; DN4, Douleur Neuropathique en 4 questions; DPN, diabetic polyneuropathy; eGFR, estimated glomerular filtration rate; F, female; HbA1c, glycated haemoglobin; HDL, high-density lipoprotein; M, male; MDNS, Michigan Diabetic Neuropathy Score; MNSI-Q, Michigan Neuropathy Screening Instrument Questionnaire; VPT, vibration perception threshold.

Values are presented as number (%), and as mean ± standard deviation or median (IQR) according to their normal or non-normal distribution.

Supplementary Table 2. Change (Δ) from follow-up to baseline in demographic, clinical and neurologic characteristics of participants who progressed, were unchanged or regressed.

Δ (FU-baseline) of variables	Progressors (P) N. 16	Unchanged (U) N. 20	Regressors (R) N. 7	P P vs U	P P vs R	P U vs R
Δ age (years)	6.44 ± 3.12	5.45 ± 2.46	4.29 ± 2.29	0.526	0.197	0.593
Δ duration (years)	5.00 (4.00)	5.35 (3.00)	4.00 (1.50)	1.000	1.000	1.000
Δ BMI (kg/m^2)	0.59 ± 1.83	0.74 ± 1.92	-0.75 ± 1.68	0.968	0.255	0.169
Δ HbA1c (%)	-0.10 ± 1.88	-0.75 ± 1.09	0.55 ± 1.01	0.401	0.617	0.127
Δ HbA1c (mmol/mol)	-0.93 ± 20.09	-8.25 ± 11.80	6.50 ± 10.29	0.363	0.577	0.099
Δ total cholesterol (mg/dl)	2.20 ± 19.08	10.39 ± 30.82	21.60 ± 17.16	0.711	0.379	0.678
Δ HDL (mg/dl)	-2.20 ± 12.04	8.56 ± 9.08	12.60 ± 8.11	**0.013**	**0.029**	0.703
Δ LDL (mg/dl)	1.17 ± 20.87	2.72 ± 23.72	0.64 ± 19.64	0.983	0.999	0.982
Δ triglycerides (mg/dl)	12.00 (71.75)	1.00 (27.75)	0.00 (23.00)	1.000	1.000	1.000
Δ eGFR (ml/min/1.73 m^2)	-5.13 ± 25.33	-4.77 ± 19.28	11.22 ± 17.12	0.999	0.329	0.316
Δ systolic BP (mmHg)	-1.17 ± 20.83	12.95 ± 14.84	-2.83 ± 19.24	0.066	0.980	0.153
Δ diastolic BP (mmHg)	1.13 ± 14.50	5.25 ± 12.83	6.67 ± 15.95	0.664	0.691	0.974
Δ DN4	0.00 (1.00)	0.00 (0.00)	0.00 (2.50)	0.536	0.557	0.937
Δ MNSI-Q	0.00 (2.25)	0.00 (1.00)	-2.00 (3.00)	0.583	0.086	0.131
Δ MDNS	3.31 ± 4.50	1.10 ± 2.53	-0.86 ± 4.18	0.179	**0.040**	0.446
Δ VPT hallux (Volt)	7.80 (6.67)	4.30 (6.87)	-1.08 (6.13)	0.087	**<0.001**	**0.025**
Δ VPT lateral malleolus (Volt)	6.70 (3.93)	4.75 (9.49)	2.15 (8.51)	0.200	0.200	0.515
Δ cold thermal threshold (°C)	-3.23 (18.14)	-0.10 (2.80)	2.30 (7.95)	0.063	**0.023**	0.271
Δ warm thermal threshold (°C)	2.27 ± 5.98	1.48 ± 3.44	-3.57 ± 7.24	0.906	0.069	0.116

Group comparisons: ANOVA with Tukey post-hoc test (normally distributed data); Kruskal-Wallis test with Dunn's post-hoc test and Holm correction for multiple comparisons (non-normally distributed data).
Abbreviations: BMI, body mass index; BP, blood pressure; Δ, change ; DN4, Douleur Neuropathique en 4 questions; FU, follow-up; HbA1c, glycated hemoglobin; MDNS, Michigan Diabetic Neuropathy Score; MNSI-Q, Michigan neuropathy screening instrument-questionnaire; VPT, vibration perception threshold.
Values are presented as number (%), and as mean ± standard deviation or median (interquartile range) according to their normal or non-normal distribution. Statistically significant P-values (p<0.05) are shown in bold.

Supplementary Figure 1. Definition of DPN Regressor, Progressor and Unchanged.

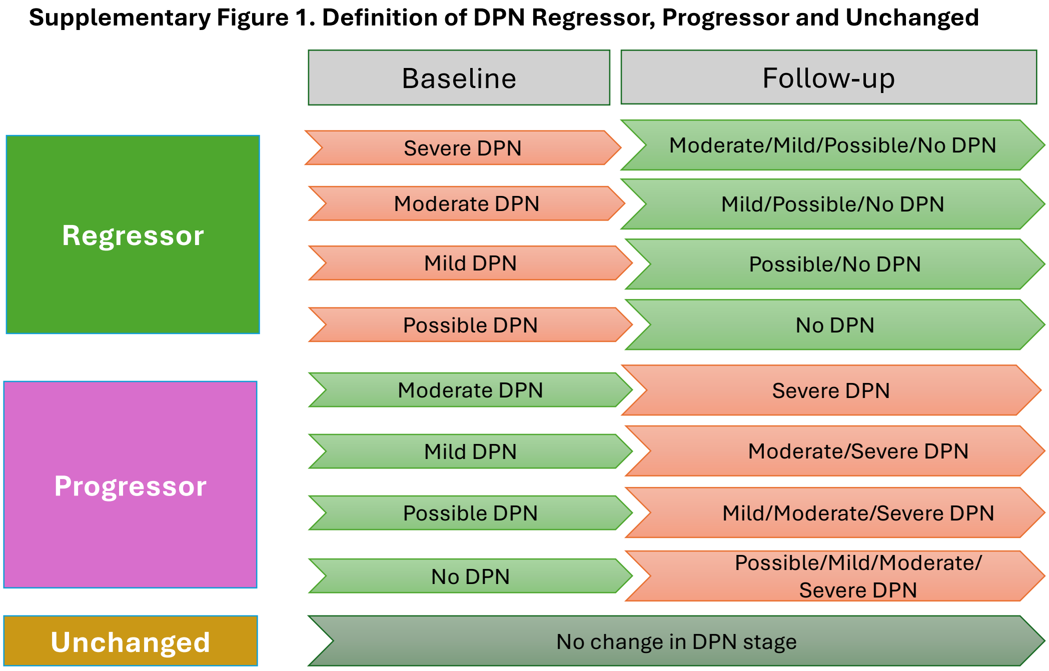

Supplement: Supplementary file 1 — Supplementary Material 1 [file 12020_2026_4613_MOESM1_ESM.pdf]
